# Twin pregnancy after kidney transplantation: case report and systematic review

**DOI:** 10.1590/2175-8239-JBN-2020-0016

**Published:** 2020-07-15

**Authors:** Marcos Vinicius de Sousa, José Paulo de Siqueira Guida, Fernanda Garanhani de Castro Surita, Mary Angela Parpinelli, Maria Laura Costa do Nascimento, Marilda Mazzali

**Affiliations:** 1 Universidade Estadual de Campinas, Faculdade de Ciências Médicas, Departamento de Clínica Médica, Divisão de Nefrologia, Unidade de Transplante Renal, Laboratório de Investigação em Transplante, Campinas, SP, Brasil.; 2 Universidade Estadual de Campinas, Faculdade de Ciências Médicas, Departamento de Tocoginecologia, Campinas, SP, Brasil.

**Keywords:** Transplantation, Pregnancy, Pregnancy, Twin, Proteinuria, Transplante, Gravidez, Gravidez de Gêmeos, Proteinúria

## Abstract

**Background:**

Kidney transplantation is associated with fertility restoration in more than 50% of women with chronic kidney disease. Pregnancy after transplantation may affect women’s health and fetal development, with higher rates of abortion, fetal growth restriction, and neonatal deaths. Twin pregnancy is a condition of high-risk for adverse maternal and perinatal outcomes, and its occurrence in women with previous kidney transplantation is rare.

**Case:**

32-year-old woman, recipient of living donor kidney transplantation, with a history of one pregnancy prior to transplantation, with current normal allograft function and no use of contraceptive method. At ten weeks of amenorrhea, ultrasound investigation showed a dichorionic diamniotic twin pregnancy. The following evaluation showed Chiari type II features in one fetus, and no detectable abnormality in the other one. There was appropriate blood pressure control with no need for an antihypertensive drug, and renal function remained normal without proteinuria. Calcium and a low dose of acetylsalicylic acid were used as preeclampsia prophylaxis. At 33 weeks of gestation, she presented premature rupture of membranes with spontaneous preterm labor. A cesarean section was performed due to the breech presentation of the first fetus. The patient persisted with normal graft function and without graft rejection during follow-up.

**Discussion and conclusion:**

Twin pregnancies after kidney transplantation are rare, and it is most frequently associated with preterm birth. We reported a successful twin pregnancy after kidney transplantation, with good perinatal and maternal outcomes, and without graft rejection or dysfunction.

## Resumo

**Introdução:**

O transplante renal está associado à restauração da fertilidade em mais de 50% das mulheres com doença renal crônica. A gravidez após o transplante pode afetar a saúde das mulheres e o desenvolvimento fetal, com taxas mais altas de aborto, restrição de crescimento fetal e óbitos neonatais. A gravidez gemelar é uma condição de alto risco para as mães e de desfechos adversos perinatais, e sua ocorrência em mulheres com transplante renal prévio é rara.

**Caso:**

mulher de 32 anos de idade, receptora de transplante de rim de doador vivo, com histórico de uma gravidez antes do transplante, com função atual normal do enxerto e sem uso de método contraceptivo. Após 10 semanas de amenorréia, a investigação por ultrassonografia mostrou uma gravidez diamniótica dicoriônica. A avaliação a seguir mostrou características de Chiari tipo II em um feto e nenhuma anormalidade detectável no outro. Houve controle adequado da pressão arterial sem necessidade de medicamento anti-hipertensivo e a função renal permaneceu normal sem proteinúria. Cálcio e uma dose baixa de ácido acetilsalicílico foram usadas como profilaxia pré-eclâmpsia. Com 33 semanas de gestação, apresentou ruptura prematura de membranas com trabalho de parto prematuro espontâneo. Uma cesariana foi realizada devido à apresentação pélvica do primeiro feto. A paciente persistiu com função normal do enxerto e sem rejeição do enxerto durante o seguimento.

**Discussão e conclusão:**

gestações gemelares após transplante renal são raras e estão mais frequentemente associadas ao parto prematuro. Relatamos uma gravidez bem-sucedida após o transplante renal, com bons desfechos perinatais e maternos e sem rejeição ou disfunção do enxerto.

## Background

Pregnancy is associated with several changes in kidney function, affecting the vascular, glomerular, and tubular components and resulting in increased renal clearance, decrease in blood pressure, and expansion of the intravascular volume 1. Advanced kidney disease disrupts the hypothalamic-pituitary-gonadal axis, reducing fertility in the absence of renal replacement therapy 2. Ovulatory cycles may begin as soon as one month after renal transplant 3, and fertility can be restored about six months after the procedure 2. Pregnancy post-transplantation may impact women’s health and fetal development, with high risk for maternal and fetal adverse events 2. The contraceptive method should be introduced before transplantation and maintained during the post-transplantation period, and it can be discontinued when it is determined that pregnancy would be relatively safe for the mother, her graft, and fetal development 3. It is recommended that women avoid pregnancy for at least one year after transplantation, due to the increased risk of potential graft dysfunction, rejection or failure, and increased risk of prematurity^3^. 

One-third of pregnancies during transplantation ends in the first trimester, due to high rates of abortion. In the remaining cases, the occurrence of neonatal death is low, with congenital disabilities occurrence similar to that observed in healthy women 4. Small-for-date babies are frequent in pregnancies of transplant recipients and pregnancies of women with hypertension 4. Prognosis of pregnancy after kidney transplantation depends on many factors, including pre-conception kidney function, previous diagnosis and adequate control of chronic hypertension and diabetes, the incidence of opportunistic infectious diseases, and the occurrence of obstetric complications^5^^-^ 7. According to the American Society of Transplantation (AST) recommendations, pregnancy is allowable in the absence of rejection within the past year, adequate and stable graft function (serum creatinine less than 1.5 mg/dL, no or minimal proteinuria less than 500 mg/24h), no acute infections that may impact fetal growth and well-being, and maintenance of adequate immunosuppression at stable dosing^3^. Twin pregnancy after kidney transplantation is rare and considered an ultra-high-risk condition 8. Our study reported a successful twin pregnancy after kidney transplantation in a referral center in Brazil and performed a literature review about this issue.

## Case Report

A 32-year-old woman, with chronic kidney disease (CKD) secondary to chronic glomerulonephritis, received a living donor kidney transplantation four years ago, with normal allograft function. The immunosuppressive therapy was tacrolimus 0.1 mg/kg bid, dose adjusted according to blood levels, azathioprine 2.0 mg/kg, and prednisone 5 mg/day. She had an obstetric history of one previous pregnancy before transplantation without complications, and currently not using any contraceptive method.

She presented at ten weeks amenorrhea, with pregnancy confirmed by serum human chorionic gonadotropin (hCG) test. There was no assisted fertilization treatment or hormonal use. Ultrasound showed a dichorionic diamniotic twin pregnancy with gestational age coincident to amenorrhea. Prenatal screening of infectious diseases and metabolic disorders at 12 weeks of gestational age showed no abnormalities. Renal function was normal (serum creatinine 0.78 mg/dL and urea level 27 mg/dL), without proteinuria in a 24-hour urine test (0.15 g). The patient presented normal parameters of blood test (hemoglobin of 12 g/dL, hematocrit 34.7%, platelets 212,000/mm^3^), normal aspartate aminotransferase and alanine aminotransferase levels (10 U/L and 13 U/L, respectively), normal serum bilirubin (1.0 mg/dL) and normal lactate dehydrogenase level (148 U/L). The patient had previous systemic arterial hypertension (SAH), but she presented normal blood pressure measurements before pregnancy in the sitting position after a five-minute rest and without antihypertensive drugs. In her first antenatal visit, she presented normal blood pressure measurement (120x80 mmHg) in the left lateral decubitus position after a five-minute rest, without any antihypertensive drugs. Because of her previous SAH, she received preeclampsia prophylaxis with a low dose of acetylsalicylic acid (100 mg) and calcium (1.5 g). The first-trimester ultrasound screening at 12 weeks was normal. The blood pressure remained stable without antihypertensive therapy. There was no change in the doses of immunosuppressive drugs, and the tacrolimus blood level remained between 3-6 ng/dL throughout the follow-up. The patient presented a reduction in the hemoglobin and hematocrit, reaching 9.6 g/dL and 29.4%, respectively, while the other laboratory parameters remained in the normal range, without the onset of proteinuria. She received iron supplementation with ferrous sulfate throughout the pregnancy.

Around 20 weeks of gestational age, an ultrasound showed multiple malformations in one of the fetuses. Such fetus had an estimated weight of 338 g, scalloping of the frontal bone (“lemon” sign), caudal displacement of the cerebellar vermis with obliteration of the cisterna magna (“banana” sign), mild ventriculomegaly, clubfeet and bifid spine with lumbosacral myelomeningocele, compatible with Chiari type II diagnosis. The other fetus had an estimated weight of 367 g without detectable abnormalities. At 28 weeks of gestational age, renal function remained stable (serum creatinine 0.79 mg/dL and urea 18mg/dL), with normal urinalysis and hematimetric parameters and negative gestational diabetes screening. 

Assessment of fetal vitality at 33 weeks of pregnancy showed a non-reassuring pattern in cardiotocography test when she was referred for hospital admission. Ultrasound revealed adequate blood flow in umbilical arteries, and preeclampsia screening was negative, with mild impairment of renal function (serum creatinine 0.92 mg/dL and urea to 26 mg/dL). On the third day of hospitalization, the patient presented premature rupture of membranes, with spontaneous onset of labor within a few hours. A cesarean section was performed due to breech presentation of the first fetus. The first newborn’s weight was 2180g with an Apgar score of 9/10 at 1 and 5 minutes after childbirth, and the second newborn had the same weight and 10/10 Apgar score.

Four days after the cesarean section, a subcutaneous hematoma was diagnosed with spontaneous regression without surgical intervention. In late puerperium, the renal function returned to pre-conception values, without proteinuria. Screening for anti-human leukocyte antigens (HLA) antibodies was negative, and allograft biopsy performed in the first year post pregnancy revealed no rejection. Both children are alive and with normal growth. The child who presented malformations underwent orthopedic surgery, and remains in follow-up, with good clinical evolution and normal physical and cognitive development.

## Methods of literature review

**Figure 1:**
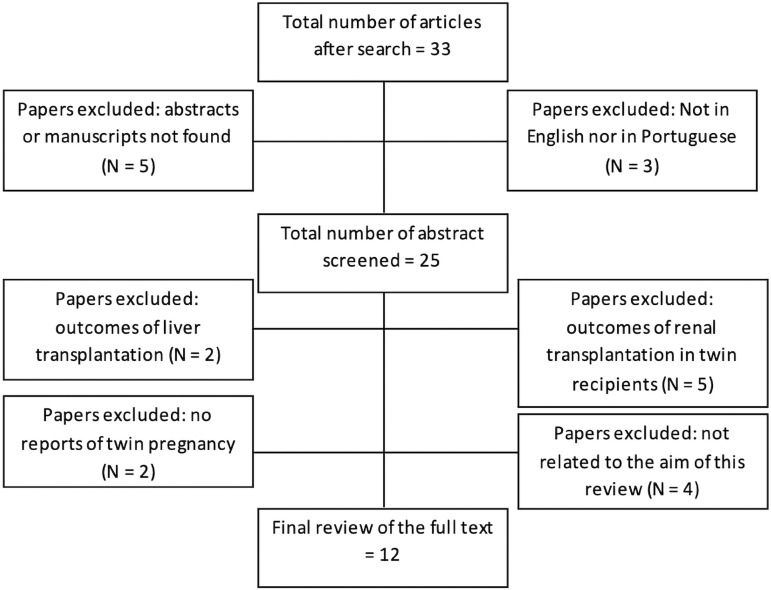
Diagram for identification of studies for the systematic review.

We performed a systematic review of the literature on PubMed. We searched from 1980 until December 2019, using the keywords “twin pregnancy” and “kidney transplantation”. Our search generated 33 articles. Three articles were excluded because they were written neither in English nor in Portuguese (two in French and one in Dutch). Of the remaining articles, five were excluded because the complete manuscript could not be found in any database (PubMed, BIREME, Scopus or Google Scholar); thirteen articles were excluded after abstracts were screened: two reported outcomes after liver transplantation; five had data about outcomes of renal transplantation in twin recipients; two articles had no reports of twin pregnancy; and four others were related to the aim of our systematic review [Figure 1]. We also reviewed studies with cases of Chiari II malformation related to the use of immunosuppressive drugs during pregnancy in this same period, but we did not find any report of this association.

## Results

We screened 12 articles; of those, 8 were case-reports, 4 were retrospective cohorts. A total of 18 twin pregnancies were reported in the literature. Results of our systematic review are shown in Table 1.

**Table 1.** Description of studies reporting the number of pregnancies, number of twin pregnancies, and complications in twin pregnancy after kidney transplantation.

Author/Year/Location	Ref.	Number of pregnancies	Number of twin pregnancies	Complications in twin pregnancy
Romão (2019), Brazil	13	2	2	Triplet pregnancy with onset of hypertension and preterm birth (34 weeks) and twin pregnancy with cesarean at 37-4/7 weeks.
Gizzo (2014), Italy	14	1	1	Preterm birth (31 weeks) after onset of hypertension and proteinuria.
Farr (2014), Austria	8	13	1	Preterm birth (30 weeks) after renal function deterioring.
Rocha (2013), Portugal	10	24	1	No report of obstetrical, fetal or allograft function complications.
Kennedy (2012), Ireland	15	27	2	One of the twin pregnancy was miscarried at 10 weeks; other twin pregnancy had miscarriage of one fetus at age of 14 weeks and birth of the second fetus at age of 30 weeks.
Cheung (2010), United Kingdon	16	1	1	Preterm birth (32 weeks) after onset of hypertension with no allograft function complication.
Gutierréz (2009), Spain	17	30	3	No report of obstetrical, fetal or allograft function complications.
Khalaf (2000), United Kingdon	18	1	1	Preterm birth (30 weeks) after spontaneous premature labour.
Furman (1999), Israel	19	2	2	Preterm birth (36 and 33 weeks), the first due to fetal growth restriction and the second due to hypertension.
Vyas (1998), United States	20	1	1	Preterm birth (32 weeks) due to hypertension; the first newborn had a cardiac malformation secondary to use of azatioprine.
Prieto (1989), Spain	21	2	2	Preterm birth (both at 35 weeks), the first due to preeclampsia and the second due to spontaneous preterm labour.
Burrows (1988), United States	22	1	1	Preterm birth (33 weeks) due to preeclampsia.

Considering only the four retrospective cohorts that we analyzed, the prevalence of twin pregnancy was 10.94%. All case-reports presented an increased risk of preterm birth, with a mean gestational age of 32.6 weeks. One of the twin pregnancies progressed to complete miscarriage, while another twin pregnancy had a miscarriage of only one of the fetus. Significant complications related to allograft function were not reported.

## Discussion

Adequate renal function (stated as creatinine levels lower than 1 mg/dL) is the main predictor of positive outcomes in pregnancies after kidney transplantation^4^^,^ 9. Physiological increase of blood volume and glomerular filtration rate may reduce serum levels of creatinine, which impairs its use as the only renal function marker during pregnancy 10. Changes in renal function occur in 10-18% of pregnancies after kidney transplantation, and early renal assessment and adequate treatment of the identified conditions are important in this population 7. Progression of renal function distress is highly associated with the onset of obstetric complications like preeclampsia or HELLP syndrome 4. Preterm birth is the most frequent complication associated with twin pregnancy, and all cases reported in our review were preterm 11.

Chronic hypertension and diabetes are frequently associated with CKD and may remain after kidney transplantation, which also affects pregnancy outcomes. The blood pressure target is lower than in general pregnancy, and it should be kept lower than 130x80 mmHg 2 ^,^ 10. Inflammatory activity in endothelium raises the risk of adverse effects during pregnancy, and the use of antihypertensive drugs may impact placental vascularization, with a higher occurrence of fetal growth restriction in this population 4. Due to the risk of teratogen effects of many antihypertensive drugs (angiotensin-converting enzyme inhibitors and angiotensin receptor blockers), the therapeutic options for hypertension management are restricted and more challenging 2.

Most immunosuppressive drugs may cross the placental barrier, reaching fetal circulation. Calcineurin inhibitors, including cyclosporine and tacrolimus, are not associated with congenital disabilities 4. Considering this class of drugs, the use of tacrolimus reduced the occurrence of preeclampsia when compared with cyclosporine 4. Considering the antiproliferative drugs, mycophenolate is contraindicated in pregnant women because of its association with an increased risk of miscarriage during the first trimester and many possible malformations, including ears, limbs, heart, esophagus or kidney, and deformations in the upper oral tract, such as cleft lip and palate 4. Azathioprine is not associated with birth defects, although it can cause neonatal leukopenia in the first year of life^4^. There is limited knowledge about the potential side effects of sirolimus or everolimus in pregnancy, and they are considered contraindicated during pregnancy since its antiproliferative effect might harm the fetus and disturb the development of the unborn 4. Corticoids increase the risk of both hypertension and gestational diabetes. Daily use of more than 20 mg of prednisone may increase the risk of opportunistic infections and preterm labor. Long term use of low doses of steroids are not associated with birth defects but can be associated with thymus hypoplasia in the newborn 4 ^,^ 12. 

The effect of pregnancy on immune status is controversial. There is a theoretical risk of sensitization by paternal HLA presented by the fetus. However, the occurrence of rejection during pregnancy is low due to the tolerance mechanism like HLA-G molecules, which inhibit T-lymphocytes, natural killer cells, and antigen-presenting cells 4. 

The diagnosis of acute rejection is obtained through graft biopsy, but it is usually not possible during pregnancy due to the risks associated with the procedure. In suspected cases of acute rejection, high doses of corticoids may be indicated, although the safety of use of lymphocytes depleting or immunoglobulins during pregnancy is unknown 2 ^,^ 7. 

Kidney allograft is usually implanted in the iliac fossa, with no mechanical influence, and under the uterus. Also, the allograft is not an obstacle to surgical delivery, and the mode of delivery may follow an obstetric indication, however, pregnancy should not exceed the 40^th^ week 3 ^,^ 7 ^,^ 12.

## Conclusion

Kidney transplantation increases obstetrical risks, and pregnancy may be planned and followed by multidisciplinary antenatal care. Contraceptive methods are essential in the pre- and post-transplant period. The gestation in transplant recipient women should be planned, and a multidisciplinary evaluation is crucial, with adequate treatment of comorbidities and adjustment of immunosuppressive therapy. Preterm birth is the most frequent complication associated with a twin pregnancy. Adequate renal function is the main predictor of good outcomes in post-transplant pregnancy, and a frequent renal function evaluation is mandatory. After pregnancy, the majority of women recover previous renal function, and immunosuppressive drugs must be adjusted.
